# Opposing effects of childhood abuse and neglect on neural performance monitoring

**DOI:** 10.1162/IMAG.a.1439

**Published:** 2026-11-04

**Authors:** Esther M. Robins, Zhiwei Zhou, Douglas J. Angus, Dennis Golm, Nicholas J. Kelley

**Affiliations:** Centre for Research on Self and Identity, School of Psychology, University of Southampton, Southampton, United Kingdom; School of Psychology, Bond University, Robina, Australia

**Keywords:** childhood adversity, abuse, neglect, error-related negativity, frontal midline theta

## Abstract

The dimensional model of adversity proposes that threat (abuse) and deprivation (neglect) exert distinct effects on neurocognitive development. We tested whether these dimensions show opposing associations with neural indices of performance monitoring in a sample of 782 young adults from the CoScience EEG-Personality Project. Participants completed the Childhood Trauma Questionnaire alongside an Eriksen flanker task while electroencephalography was recorded. Performance monitoring was indexed by the error-related negativity and frontal midline theta activity. Consistent with preregistered hypotheses, abuse and neglect showed opposing associations with both neural measures. Higher abuse was associated with enhanced error-related negativity but not theta power, whereas higher neglect was associated with attenuated error-related negativity and reduced theta power. Exploratory analyses indicated that these associations were moderated by sex, with stronger effects observed in males. These findings are consistent with the distinction between threat- and deprivation-related adversity and suggest that different forms of childhood adversity may be linked to variation in neurocognitive processes underlying cognitive control. However, the cross-sectional and retrospective nature of the measures limits causal inference and may introduce reporting biases.

## 1 Introduction

Early childhood experiences play a central role in shaping mental health and well-being across the lifespan. Child adversity can be conceptualized in terms of two core dimensions: abuse and neglect. Abuse involves the presence of harmful acts, such as verbal or physical aggression, whereas neglect reflects the absence of essential care, including access to food, shelter, or attention (National Institute for Health and Care Excellence [NICE], 2017). These experiences represent deviations from the expected environment required for normative development (McLaughlin, 2016) and consequently are associated with alterations in brain and cognitive development that persist into adulthood (Cao et al., 2025; Felitti et al., 2019; Lynch & Widom, 2022; Wong et al., 2025). Although childhood adversity can be distinguished into specific forms and dimensions, much of the literature has examined maltreatment in broad or overly simplified ways, often collapsing exposure into global categories of “any maltreatment” (Rivera et al., 2017). As a result, it remains unclear whether abuse and neglect are associated with similar or distinct neurocognitive profiles. In the current study, we examine how abuse and neglect are differentially associated with neural indices of performance monitoring in a large sample of young adults.

### 1.1 Performance monitoring and the dimensional model of adversity

The dimensional model of adversity (McLaughlin et al., 2014, 2019) proposes that threat and deprivation reflect distinct dimensions of experience with partially separable neurocognitive consequences. Threat-related adversity, such as abuse and violence exposure, is expected to alter neural systems involved in salience detection (Goetschius et al., 2020; Weissman et al., 2020), aversive learning (DeCross et al., 2022; Machlin et al., 2024), and emotion regulation (Gruhn et al., 2024; Jenness et al., 2021), consistent with heightened vigilance to cues signalling potential harm. In contrast, deprivation-related adversity, such as neglect, is expected to more strongly affect neural systems supporting cognitive development, including executive functioning and higher-order cognition (Schäfer et al., 2023; Sheridan et al., 2017; Wang et al., 2026), as reduced social and cognitive input limits opportunities for learning and environmental feedback. These predictions suggest that abuse and neglect may have different implications for performance monitoring, particularly processes involved in detecting salient mistakes and engaging control processes more generally.

Performance monitoring may be especially relevant in this context because it depends on both rapid salience-sensitive responses to mistakes and broader control-related processes that support behavioural adjustment. Neural activity within the anterior cingulate cortex (ACC) has been consistently implicated in these processes (Holroyd & Verguts, 2021). Two commonly used neural indices of performance monitoring, localized in part to the ACC, are the error-related negativity (ERN), a rapid frontocentral response following errors thought to reflect early error detection (Becker et al., 2014; Botvinick et al., 2001; Carter et al., 1998; Cole et al., 2024; Imburgio et al., 2020; Umemoto et al., 2017) and frontal midline theta, which reflects more sustained control-related processes engaged during performance monitoring (Cavanagh & Frank, 2014; Cavanagh et al., 2012; Chevalier et al., 2021). If threat-related adversity calibrates neural systems towards heightened sensitivity to potential harm, it may be associated with enhanced ERN and theta responses. In contrast, if deprivation-related adversity disrupts the development of systems that rely on environmental input and feedback, it may be associated with attenuated performance-monitoring signals.

### 1.2 Childhood adversity and neural indices of performance monitoring

A growing body of research has examined the relationship between childhood adversity and neural indices of performance monitoring, although findings remain inconsistent. For the ERN, some studies report enhanced (more negative) amplitudes associated with adversity, interpreted as heightened sensitivity to errors and increased allocation of cognitive control (Cole et al., 2023; Compton et al., 2024; Lackner et al., 2018; Wu et al., 2021). In contrast, other studies report attenuated (less negative) ERN amplitudes associated with adversity, suggesting reduced error sensitivity and poorer behavioural regulation (Letkiewicz et al., 2023; Tabachnick et al., 2018; Xue et al., 2017). Evidence linking childhood adversity to frontal midline theta is limited and mixed. One study found that greater early childhood adversity predicted greater resting theta power at age 8 years. A task-based longitudinal study found that institutional rearing predicted reduced frontal midline theta during a cognitive control task (i.e., an emotionally neutral Go/No-Go task) in adolescence (Buzzell et al., 2020). More recently, among children aged 9–13 years, adversity over the last 6 months was associated with reduced frontal midline theta during a Zoo-themed emotionally neutral Go/No-Go task, although only among children with lower levels of early life adversity (Kavanaugh et al., 2024). One potential explanation for these inconsistencies is that many studies rely on cumulative measures of adversity. If abuse and neglect exert distinct or even opposing effects on performance monitoring, aggregating them into a single measure may obscure their potentially divergent associations.

Although the ERN and error-related theta are related markers of performance monitoring and are both generated primarily within the medial frontal cortex, accumulating evidence suggests that they reflect partially distinct cognitive processes. The ERN is a transient event-related potential that occurs immediately following an erroneous response and is thought to index the rapid detection and evaluation of errors (Gehring et al., 1993; Holroyd & Coles, 2002). In contrast, frontal midline theta power reflects the recruitment and coordination of cognitive control processes that support behavioural adaptation following errors (Cavanagh & Frank, 2014). Although the ERN is accompanied by increases in theta activity, the two measures are only moderately associated and can show dissociable relationships with behaviour, psychopathology, and individual differences, suggesting that they provide complementary information about performance monitoring (Cavanagh & Frank, 2014; Cohen, 2014b). Examining both measures may, therefore, provide a more comprehensive understanding of how childhood adversity influences performance monitoring.

### 1.3 The current study

In the current study, we adopt a dimensional approach to examine how abuse and neglect are differentially associated with neural indices of performance monitoring. The present study involves a secondary analysis of data from the CoScience EEG-Personality Project (Paul et al., 2022), in which participants completed a retrospective measure of childhood adversity (Bernstein et al., 2003; Klinitzke et al., 2012) alongside an Eriksen flanker task (Eriksen & Eriksen, 1974) during EEG recording. Study materials are described in Paul et al. (2022). Guided by the dimensional model of adversity, we hypothesized that abuse would be associated with enhanced performance monitoring, reflected in a larger (more negative) ERN and increased frontal midline theta activity. In contrast, we hypothesized that neglect would be associated with attenuated performance monitoring, reflected in a reduced (less negative) ERN and decreased frontal midline theta activity.

Although evidence regarding sex differences in the effects of childhood adversity on performance monitoring is limited, previous research suggests that the neurodevelopmental consequences of childhood adversity may differ between males and females, with males often showing greater vulnerability to externalizing and neurocognitive outcomes following adverse experiences (Pechtel & Pizzagalli, 2011; Teicher et al., 2016). Given these findings, and the balanced representation of males and females in the present sample, we conducted exploratory moderation analyses to determine whether associations between childhood adversity and neural indices of performance monitoring varied by sex.

## 2 Method

### 2.1 Participants

This study is a secondary data analysis of the CoScience EEG-Personality Project (Paul et al., 2022). From the original sample (*N* = 822), we first excluded participants with missing childhood trauma data (*n* = 14), then those with excessive EEG artefact resulting in an insufficient number of usable trials after preprocessing (*n* = 24), and finally those fewer than six errors (Olvet & Hajcak, 2009; *n* = 2). Ethnicity information was available for 808 participants and missing for 14 participants. Among those with available ethnicity data, 736 identified as European (91.1%), 11 as Arabic (1.4%), 7 as African (0.9%), 29 as Asian (3.6%), 6 as Hispanic (0.7%), and 19 as another ethnicity (2.4%). The final analytic sample comprised 782 participants: 715 identified as European (91.4%), 11 as Arabic (1.4%), 6 as African (0.8%), 28 as Asian (3.6%), 6 as Hispanic (0.8%), and 16 as another ethnicity (2.0%).

These exclusion criteria were pre-registered. The final analytic sample comprised 782 participants from the following universities: Bonn (*n* = 81), Dresden (*n* = 81), Giessen (*n* = 79), Hamburg (Riesel lab; *n* = 72), Hamburg (Wacker lab; *n* = 77), Koeln (*n* = 74), Marburg (*n* = 77), Oldenburg (*n* = 80), Osnabrueck (*n* = 84), and Wuerzburg (*n* = 77). This sample size conferred 87% power to detect small bivariate associations (*r* = .11; Faul et al., 2007). Participants were between 18 and 30 years old (*M* = 23.36, *SD* = 2.95) and identified as female (*n* = 396, 50.6%) or male (*n* = 386, 49.4%).

### 2.2 Procedure

#### 2.2.1 Recruitment and screening

Participants were recruited through online and offline postings on university campuses, university-related platforms, and social networks. They were eligible to participate if they were between 18 and 30 years old, heterosexual,^1^ right-handed, fluent in German, and able to provide informed consent. Additionally, participants were excluded if they reported any current or past physical or psychological disorder, use of prescription medication, drug abuse, regular smoking, or dreadlocks, as this hairstyle can impede consistent electrode–scalp contact and compromise EEG signal quality. For completion, participants were reimbursed with €10 per hour or course credits plus predetermined in-task winnings of €12.50. The study protocol was approved by the ethics committee of the German Society of Psychology. Our secondary data analysis was approved by the Psychology Ethics Committee, University of Southampton (Protocol Number 109226).

#### 2.2.2 Online assessment

After the screening process, eligible participants provided informed consent and completed demographic questions assessing sex, age, ethnicity, relationship status, employment status, job title, highest academic qualification, average gross monthly income, use of hormonal contraceptives, and handedness. Participants then completed an online assessment comprising a comprehensive battery of 18 self-report personality and individual difference questionnaires administered in a fixed order (Paul et al., 2022).

##### 2.2.2.1 Childhood trauma questionnaire

Of central relevance to the present study, the battery included the Brief Childhood Trauma Questionnaire (CTQ; Bernstein et al., 2003; Klinitzke et al., 2012), which assessed retrospectively reported adverse childhood experiences. Participants indicate the extent to which 28 statements apply to them on a scale of 1 (*Never True*) to 5 (*Very Often True*). The CTQ comprises five clinical subscales, each containing five items (which we summated to create the composite scores reported in Table 1): emotional abuse, physical abuse, sexual abuse, emotional neglect, and physical neglect. In addition, the questionnaire includes a three-item minimization/denial validity scale, which is designed to detect potential underreporting of childhood adversity and is not included in the calculation of subscale or total scores. The five clinical subscales contain emotional abuse (e.g., “People in my family said hurtful or insulting things to me”), physical abuse (“I got hit so hard by someone in my family that I had to go to see a doctor or go to the hospital”), sexual abuse (e.g., “Someone molested me.”), emotional neglect (“There was someone in my family who helped me feel that I was important or special” *Reverse Coded*) and physical neglect (e.g., “When I was growing up, I didn’t have enough to eat”). Descriptive statistics for individual CTQ subscales, CTQ abuse composite score (sum of emotional, physical, and sexual abuse), CTQ neglect composite score (sum of emotional and physical neglect), and CTQ total (sum of all CTQ abuse and CTQ neglect) in the current sample are presented in Table 1.

**Table 1.** Descriptive statistics and internal consistency for childhood trauma questionnaire scores.

CTQ measure	Observed range	*M*	*SD*	α
Emotional abuse	5–25	8.09	3.72	.83
Physical abuse	5–18	5.62	1.58	.72
Sexual abuse	5–25	6.30	3.43	.88
Emotional neglect	5–20	5.58	1.97	.90
Physical neglect	5–18	6.68	2.56	.44
CTQ abuse	15–54	19.30	5.88	.86
CTQ neglect	10–42	16.03	6.15	.83
Total CTQ	25–83	35.93	10.50	.90

The CTQ abuse score (emotional, physical, and sexual abuse) ranged from 15 to 54 (*M* = 19.30, *SD* = 5.88, α = .86) and the CTQ neglect score (emotional and physical neglect) ranged from 10 to 42 (*M* = 16.03, *SD* = 6.15, α = .83). The total CTQ score ranged from 25 to 83 (*M* = 35.93, *SD* = 10.50, α = .90).

Using established CTQ cut-offs, the prevalence of childhood adversity in the sample (*N* = 711) was as follows: 13.1% of participants met criteria for emotional abuse, 4.1% for physical abuse, and 7.9% for sexual abuse. For neglect, 13.9% met criteria for emotional neglect and 11.7% for physical neglect.

#### 2.2.3 Laboratory session

Participants completed a single laboratory session lasting approximately 5 hours, during which continuous EEG and ECG were recorded (see Paul et al. (2022) for full procedural details). Upon arrival, participants reported recent sleep, caffeine, alcohol, and food intake, and female participants additionally reported menstrual cycle phase. Brief assessments of fluid and crystallized intelligence were administered prior to the main task battery.^2^

##### 2.2.3.1 Eriksen flanker task

As part of the main task battery,^3^ participants completed an Eriksen flanker task (Eriksen & Eriksen, 1974). On each trial, participants responded with their right hand to a centrally presented target letter while ignoring flanking letters. When responses exceeded an individualized latency threshold determined during practice, written feedback indicated that the response was too slow. Two manipulations were implemented. First, target–flanker congruency was manipulated across four levels: 100% congruent (e.g., SSSSSSS), 67% congruent (e.g., HSSSSSH), 33% congruent (e.g., HHSSSHH), and 0% congruent (e.g., HHHSHHH). Second, social evaluation was manipulated across blocks such that the experimenter was present during three blocks and absent during three blocks; block order was counterbalanced across participants. For the present analyses, trials were collapsed across social evaluation and congruency conditions, yielding a 2 (response: correct vs. incorrect) × 2 (congruency: congruent vs. incongruent) design. Error-related neural activity was quantified from this task as described below.

##### 2.2.3.2 EEG recording and data reduction

We preprocessed EEG data in Python 3.12.2 4, using MNE 1.8.0. First, we resampled to 250 Hz and applied a 1–40 Hz band-pass filter. A 10–20 montage was utilized and we rereferenced to the average. We used independent component analysis (ICA) to address ocular artefacts with 20 components via FastICA (Hyvärinen & Oja, 2000). We used MNE’s built in detection algorithm to identify EOG-related components. We extracted event codes from annotation labels and using these, continuous data were epoched from −2000 to 2000 ms. Baseline correction was applied to each response-locked epoch using the mean signal amplitude from −200 to 0 ms relative to the response. We used the RANSAC algorithm (autoreject; Jas et al., 2017) to detect bad channels and subsequently interpolated them within the epoched data. Epoch level artefact rejection was employed through a two-stage process: (1) application of a fixed peak-to-peak threshold (150 µV), followed by (2) a z-score-based criterion, where peak-to-peak amplitude for each epoch and channel was computed, z-scored across epochs within each channel, and any epoch with a maximum absolute z-score exceeding 2 was excluded. On average, 67.80% (*SD* = 23.60) of trials were retained following artefact rejection, corresponding to an average of 565 trials before and 383 trials after preprocessing per participant.

We extracted response-locked ERP amplitudes (ERN, CRN) in both the 0–50 ms and the 0–100 ms time window post event code from FCz. The incorrect-minus-correct difference wave showed a clear frontocentral negativity during the ERN peak window of 54–74 ms. Both FCz and Cz were located within this negative field, with the distribution appearing maximal closer to FCz, supporting the use of FCz for ERN quantification (see Fig. 1). Frontal midline theta was calculated using time–frequency decomposition using complex Morlet wavelets on the EEG signal at electrode FCz. Theta power was defined within the 4–8 Hz range. Primary theta responses were identified via automatic peak detection applied to the mean theta power time course averaged across all conditions. Peak identification was restricted to the 250–550 ms interval post-event, corresponding to the expected period of maximal midfrontal theta during performance monitoring. To reduce bias due to latency differences across conditions, a 40 ms peak-detection window was centred on the automatically identified group-level peak. Theta amplitude analyses were subsequently conducted within this fixed window for each condition and participant.

**Fig. 1.**
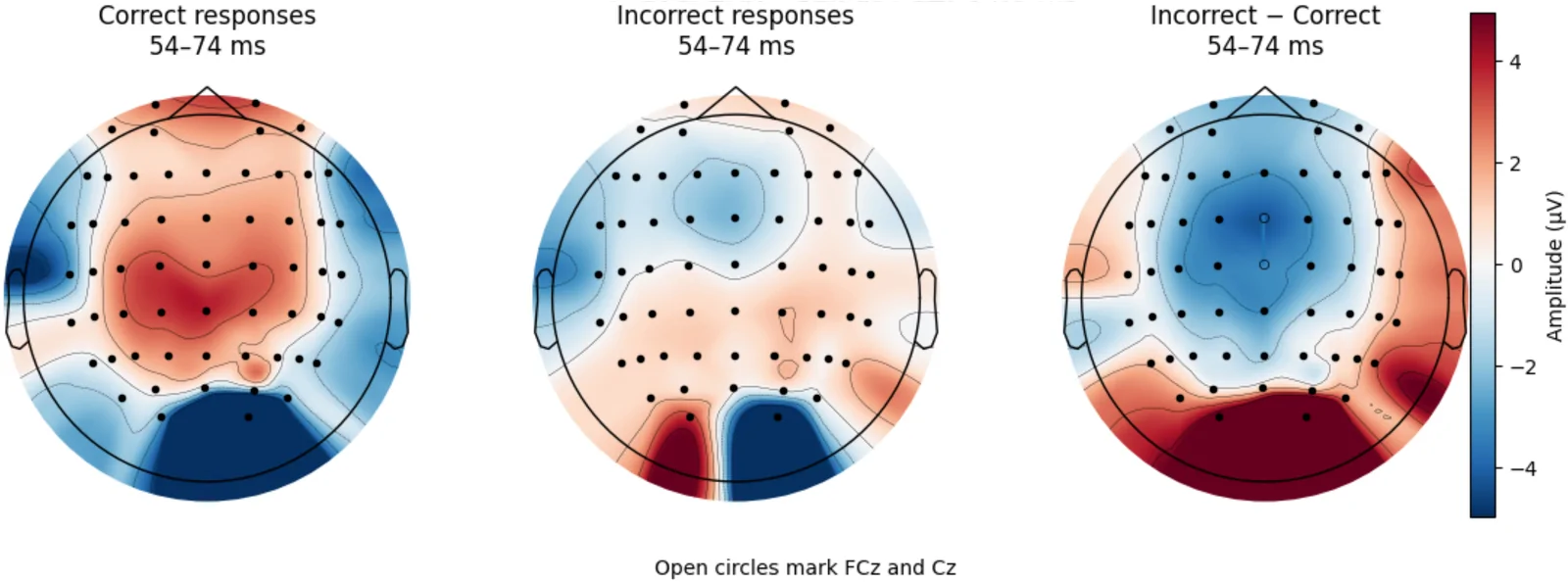
Response-locked ERP scalp distributions during the ERN peak (54–74 ms).

#### 2.2.4 Research transparency

Artificial intelligence tools were used to assist with language editing and to support code development. However, all analyses, methodological decisions, and interpretations were conducted and verified by the authors. The study was preregistered prior to data access (https://osf.io/c7pa4), including the study hypotheses and analysis plan. Raw data are not publicly available; however, researchers can apply for access via the CoScience data access process (Paul et al., 2022). All derived data and analysis scripts necessary to reproduce the reported results are publicly available on the Open Science Framework (https://osf.io/c7pa4).

## 3 Results

### 3.1 Preliminary analyses

#### 3.1.1 Error-related negativity

Visual inspection of the grand average waveforms indicated that the ERN peak extended across the 0–100 ms window following the response (see Fig. 2), rather than being fully captured within the narrower 0–50 ms window.^4^ Accordingly, ERN amplitude was quantified as the mean amplitude from 0 to 100 ms following response for all primary analyses. We conducted a repeated-measures ANOVA and observed a significant main effect of response such that amplitudes were larger (i.e., more negative) on incorrect (*M* = −1.49 µV) than on correct (*M* = 1.66 µV) responses, *F*(1, 722) = 159.92, *p* < .001. There was no significant main effect of congruency, *F*(1, 722) = 0.57, *p* = .449, nor was there a significant response × congruency interaction, *F*(1, 722) = 0.94, *p* = .333. Thus, ERN amplitude was sensitive to response accuracy but not to stimulus congruency. This is consistent with prior work showing that conflict manipulations in the flanker task do not always produce reliable differences in ERP components (Kałamała et al., 2017). Given the absence of congruency effects, subsequent analyses focused on response type.

**Fig. 2.**
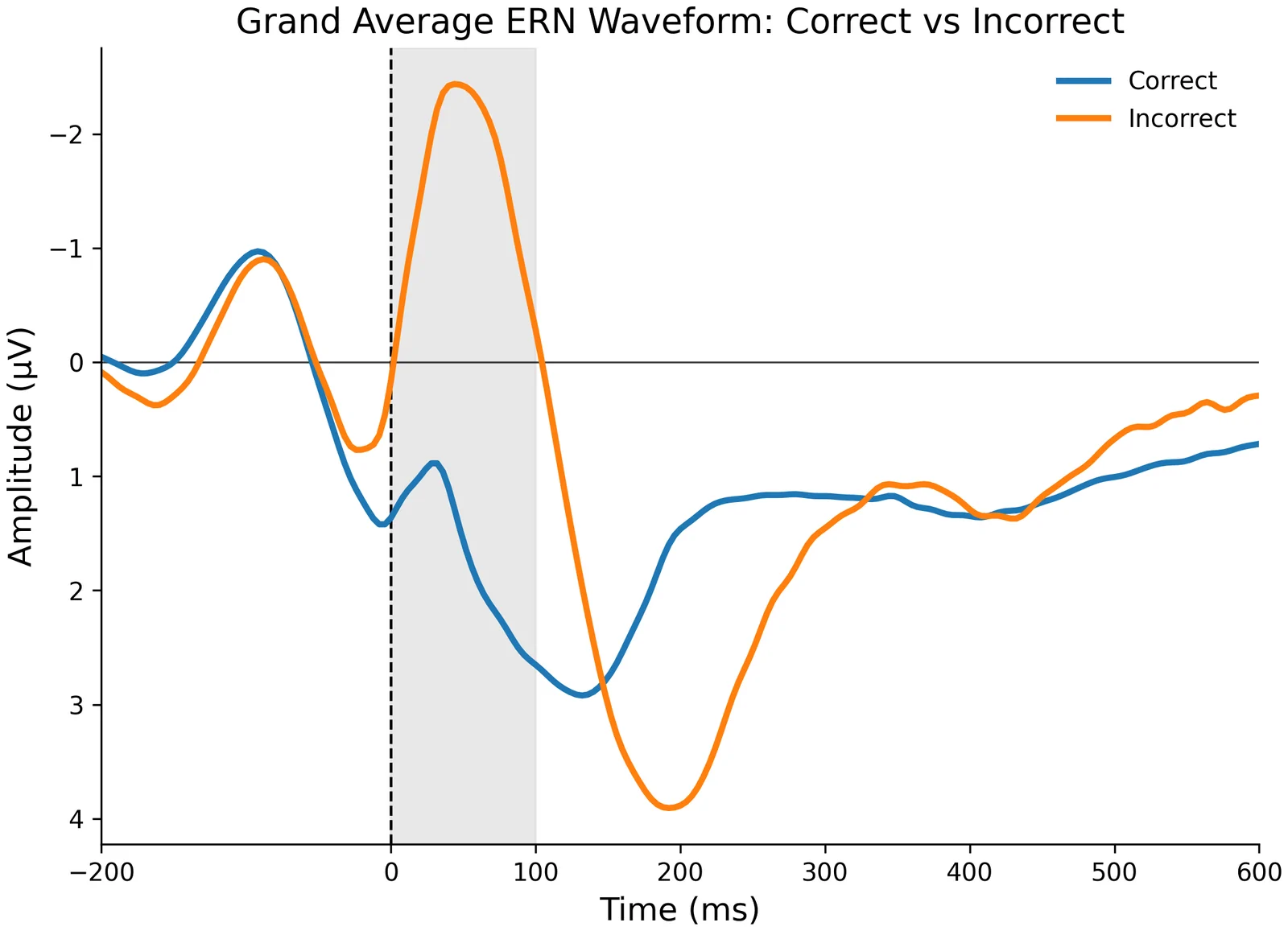
Grand average ERN difference amplitude (-200 to 600 ms) waveform at FCz.

#### 3.1.2 Frontal midline theta

We conducted a 2 (response: correct, incorrect) × 2 (congruency: congruent, incongruent) repeated-measures ANOVA examining frontal midline theta power (4–8 Hz), quantified within the 40 ms window centred on the group-level theta peak identified between 250 and 550 ms post-response. There was no significant main effect of response, *F*(1, 722) = 0.13, *p *= .715, indicating that theta power did not differ between correct and incorrect responses. There was also no significant main effect of congruency, *F*(1, 722) = 0.07, *p *= .795, nor was there a significant response × congruency interaction, *F*(1, 722) = 2.00, *p *= .158. Thus, theta power did not significantly vary as a function of response accuracy or stimulus congruency. Given the absence of reliable congruency effects, subsequent analyses were collapsed across congruency conditions and response types.

### 3.2 Bivariate correlations

There was a significant positive correlation between ERN and error-related theta, *r*(708) = .16, *p* < .001. However, the association was small, with the two measures sharing approximately 2.5% of their variance. When examined separately by trial type, the congruent error effects showed a small negative correlation, *r*(708) = −.11, *p* = .004, whereas the incongruent error effects were not significantly correlated, *r*(708) = .05, *p* = .203.

As expected, CTQ abuse and CTQ neglect were significantly positively correlated, *r*(708) = .65, *p* < .001, indicating substantial co-occurrence of the two adversity dimensions within the present sample. Neither abuse nor neglect was significantly associated with overall task accuracy, error rate, number of errors, post-error accuracy, or the flanker congruency effect (all |*r*|s < .04, all *p’*s > .30). A small positive correlation was observed between abuse and post-error slowing, *r*(708) = .09, *p* = .023, but this did not survive correction for multiple comparisons. The corresponding association with neglect was not significant, *r*(708) = .07, *p* = .078. Overall, these findings suggest that childhood adversity was not associated with substantial differences in behavioural performance on the flanker task.

### 3.3 Linear mixed-effects models

To test our preregistered hypotheses, we estimated a series of linear mixed-effects models predicting neural responses during the flanker task. We pre-registered using both Frequentist and Bayesian models for ERN and theta. ERN Bayesian models produced similar results. Theta Bayesian model effects were either non-significant or much smaller though in the same direction. This could reflect smaller effect sizes or increased noise in task-related theta compared with ERN. Bayesian models are given in Supplementary Tables S1 and S2. All analyses were conducted in *R* (Version 4.5.0; R Core Team, 2025). Data wrangling and visualization were performed using the tidyverse suite of packages (Wickham et al., 2019), including ggplot2 for plotting (Wickham, 2016) and scales for axis formatting (Wickham & Seidel, 2022). Linear mixed-effects models were fitted using lme4 (Bates et al., 2015), with statistical inference obtained via lmerTest (Kuznetsova et al., 2017). Estimated marginal means and model-based visualizations were generated using emmeans (Lenth, 2024). Model assumptions were assessed via visual inspection of residual and Q–Q plots. Although minor deviations from normality were observed in the tails of the ERN data, residuals were approximately symmetrically distributed and showed no substantial heteroscedasticity. Given the large sample size, these deviations were not considered to meaningfully impact inference. However, given the positively skewed distribution typical of EEG spectral power, theta values were log-transformed prior to analysis to stabilize variance and improve conformity with model assumptions (Box & Cox, 1964; Cohen, 2014a). We hypothesized that childhood abuse would be associated with a heightened (more negative) ERN and increased theta activity, whereas childhood neglect would be associated with a blunted (less negative) ERN and reduced theta activity. These hypotheses were operationalized as interactions between childhood adversity and trial type for ERN analyses,^5^ and as main effects for theta activity where appropriate. We further conducted exploratory analyses to examine whether sex altered the associations between childhood adversity and neural responses.

#### 3.3.1 ERN

##### 3.3.1.1 Primary model: Main effects of childhood adversity

We first fit a linear mixed-effects model to examine the extent to which CTQ abuse, CTQ neglect, and sex were associated with response-locked ERP amplitude in the 0–100 ms window following the response, and whether these associations differed by trial type. Trial type (correct vs. incorrect) was included such that the ERN was operationalized as the difference in amplitude between incorrect and correct trials. We entered CTQ abuse (z-scored) and CTQ neglect (z-scored) simultaneously into the same model as fixed effects, together with sex, allowing the unique association of each adversity dimension with ERP amplitude to be estimated while accounting for their shared variance. Interactions between trial type and each predictor were included to test whether the ERN varied as a function of childhood adversity and sex. Random intercepts were included for participant and laboratory site. These indicated minimal variability across participants (σ² = 5.01 × 10^−^¹¹ V²) and laboratories (σ² ≈ 0 V²) in response-locked ERP amplitude. Residual variance was 2.14 × 10^−9^ V². The results of the primary and secondary models are presented in Table 2.

**Table 2.** Linear mixed-effects model results for response-locked event-related potential amplitudes.

Model	Predictor	B (µV)	SE (µV)	95% CI	df	*t*	*p*
Primary model	Intercept	2.659	0.390	[1.894, 3.425]	885.6	6.82	< .001
	Response (incorrect vs. correct)	-3.659	0.298	[-4.244, -3.074]	304,259.3	-12.26	< .001
	Childhood abuse	0.793	0.363	[0.081, 1.505]	898.7	2.19	.029
	Childhood neglect	-0.615	0.362	[-1.326, 0.095]	892.5	-1.70	.090
	Sex (female vs. male)	-1.855	0.556	[-2.946, -0.765]	890.3	-3.34	< .001
	Response × childhood abuse	-0.609	0.290	[-1.178, -0.040]	304,656.1	-2.10	.036
	Response × childhood neglect	0.475	0.285	[-0.084, 1.033]	304,112.5	1.67	.096
	Response × sex	0.918	0.430	[0.074, 1.761]	304,523.3	2.13	.033
Secondary moderation model	Intercept	3.013	0.400	[2.229, 3.797]	886.0	7.54	< .001
	Response (incorrect vs. correct)	-3.857	0.307	[-4.458, -3.256]	304,413.1	-12.58	< .001
	Childhood abuse	2.995	0.714	[1.594, 4.396]	883.9	4.20	< .001
	Sex (female vs. male)	-2.080	0.556	[-3.170, -0.990]	890.7	-3.74	< .001
	Childhood neglect	-1.838	0.556	[-2.929, -0.748]	888.4	-3.31	< .001
	Response × childhood abuse	-1.871	0.537	[-2.923, -0.818]	304,550.0	-3.48	< .001
	Response × sex	1.039	0.432	[0.192, 1.887]	304,526.9	2.40	.016
	Childhood abuse × sex	-2.995	0.830	[-4.625, -1.366]	889.1	-3.61	< .001
	Response × childhood neglect	0.929	0.428	[0.090, 1.767]	303,422.4	2.17	.030
	Childhood neglect × sex	1.872	0.736	[0.427, 3.317]	891.9	2.54	.011
	Response × childhood abuse × sex	1.721	0.641	[0.465, 2.978]	304,628.3	2.68	.007
	Response × childhood Neglect × sex	-0.610	0.579	[-1.744, 0.524]	303,951.7	-1.05	.292

Incorrect responses elicited significantly more negative ERP amplitudes than correct responses, consistent with the expected ERN effect. Higher levels of CTQ abuse were associated with more positive overall ERP amplitudes across trial types, while females exhibited more negative overall ERP amplitudes than males. Importantly, the significant interaction between response and CTQ abuse indicated that increasing levels of CTQ abuse were associated with a larger (i.e., more negative) ERN. We show these interactions in Figure 3. For illustrative purposes, in Figure 4 we also report the ERN difference waveforms separately for those high (top 25%) and low (bottom 25%) in both CTQ abuse and CTQ neglect. A significant response × sex interaction further indicated that the magnitude of the ERN differed between males and females.

**Fig. 3.**
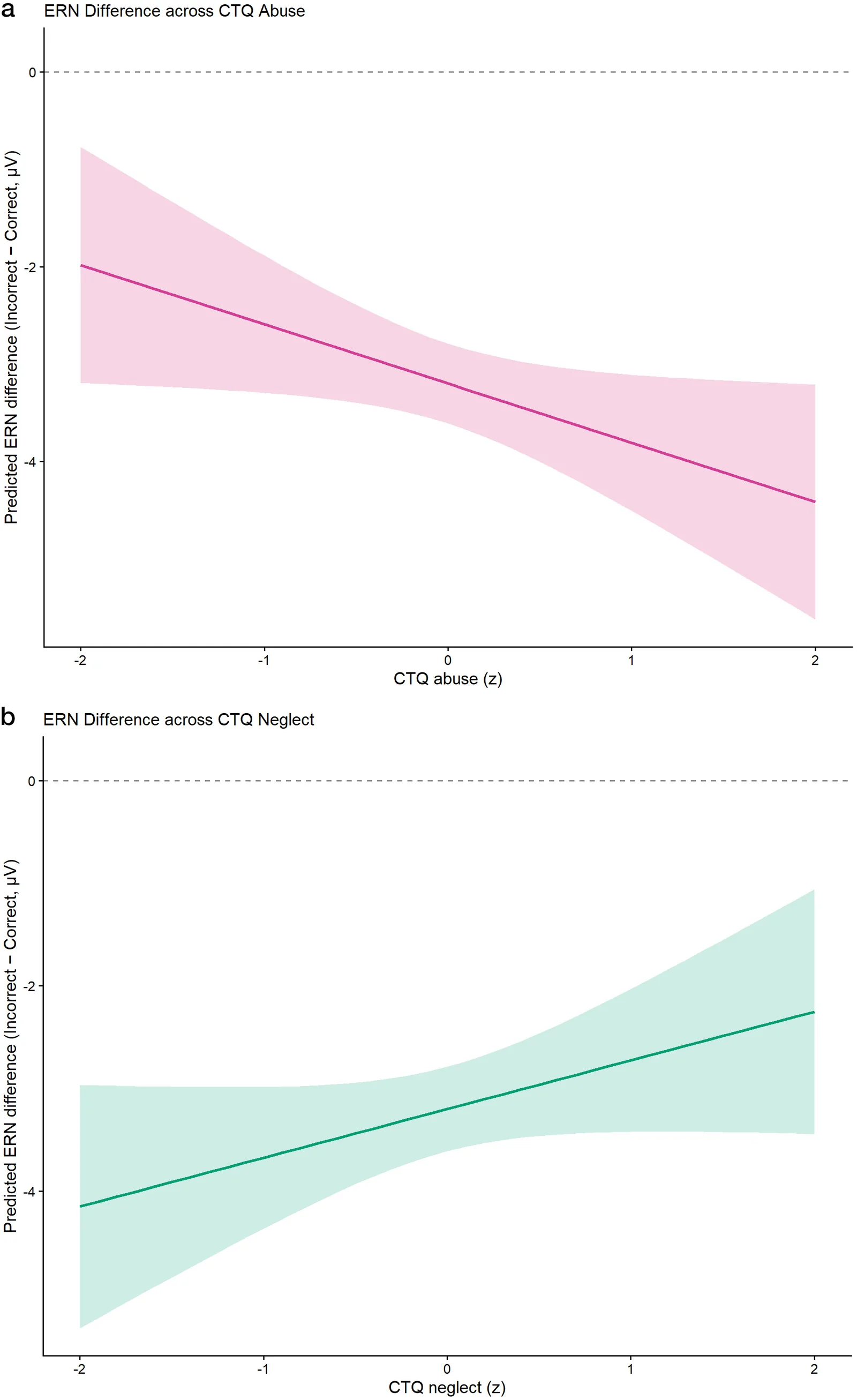
The association between ERN difference and childhood adversity.

**Fig. 4.**
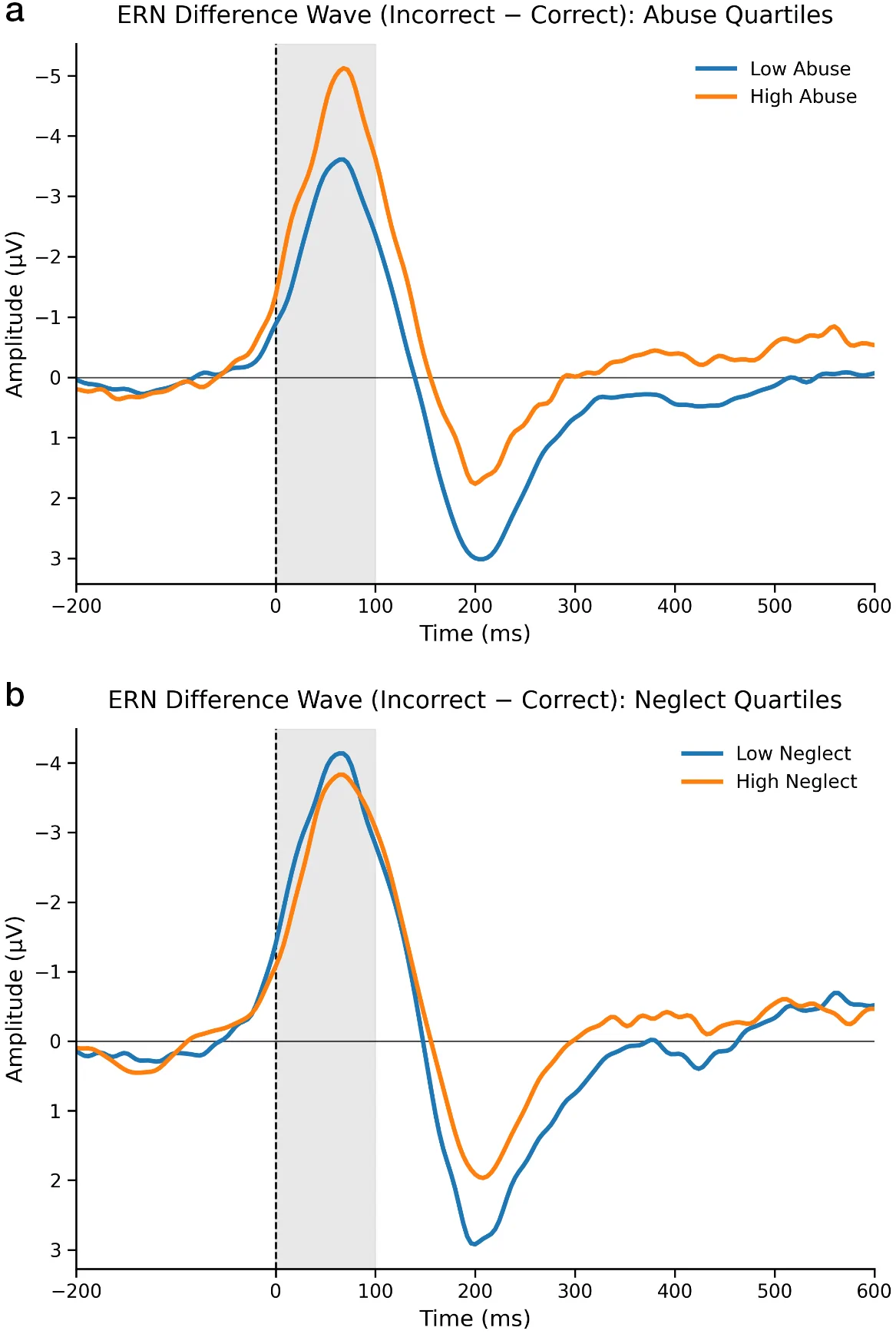
ERN difference for those high (25th percentile) and low (75th percentile) in CTQ abuse (top) and neglect (bottom).

##### 3.3.1.2 Secondary model: Moderating effects of sex

To examine whether associations between childhood adversity and the ERN differed by sex, we fitted a second linear mixed-effects model that extended the primary model by including three-way interactions between trial type, CTQ abuse, and sex, and between trial type, CTQ neglect, and sex. This model enabled us to test whether sex moderated the relationship between childhood adversity and neural indices of error monitoring while retaining the same random-effects structure as the primary model. Including these interaction terms significantly improved model fit relative to the primary model, Δχ²(4) = 18.62, *p* < .001.

As in the primary model, incorrect responses elicited significantly more negative ERP amplitudes than correct responses. Higher levels of CTQ abuse were associated with more positive overall ERP amplitudes, whereas higher levels of CTQ neglect were associated with more negative overall ERP amplitudes.

The significant response × CTQ abuse interaction indicated that increasing levels of CTQ abuse were associated with a larger (more negative) ERN, whereas the significant response × CTQ neglect interaction indicated that increasing levels of CTQ neglect were associated with a smaller (less negative) ERN. Significant interactions between sex and both CTQ abuse and CTQ neglect indicated that the associations between adversity and overall ERP amplitudes differed between males and females. Furthermore, the significant response × CTQ abuse × sex interaction demonstrated that the association between childhood abuse and ERN magnitude differed by sex. Specifically, the ERN became progressively larger (more negative) as CTQ abuse increased in males, whereas ERN amplitude remained relatively stable across levels of CTQ abuse in females. No comparable three-way interaction was observed for CTQ neglect, indicating that the relationship between neglect and ERN magnitude did not differ significantly between males and females (Fig. 5).

**Fig. 5.**
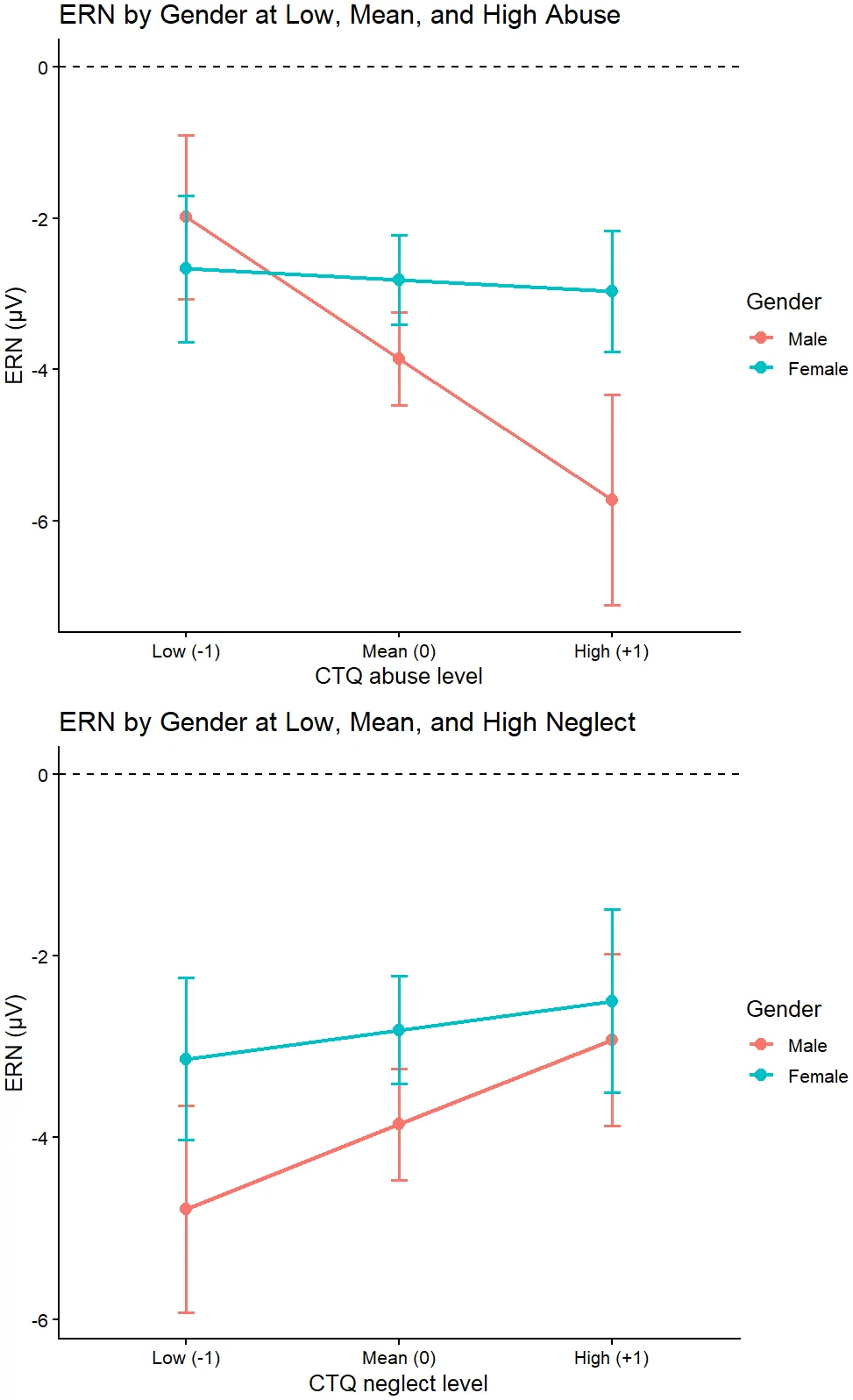
Sex moderates the association between ERN difference and childhood adversity.

Simple slopes analyses supported this interpretation. Among males, higher CTQ abuse was associated with a significantly larger (more negative) ERN, whereas this association was not significant among females. In contrast, although higher CTQ neglect was associated with a smaller (less negative) ERN in males, the corresponding simple slope was not significant in females (Table 3). These effects remained consistent when controlling for anxiety and depression (see Supplementary Table S3).

**Table 3.** Simple effects results for response-locked event-related potential amplitudes.

Predictor	Sex	Response	B	SE	95% CI	*z*	*p*
Childhood abuse	Male	Correct	2.995	0.714	[1.596, 4.394]	4.20	< .001
		Incorrect	1.124	0.818	[-0.480, 2.729]	1.37	.170
		Incorrect–correct	-1.871	0.537	[-2.923, -0.818]	-3.48	< .001
	Female	Correct	-0.000	0.424	[-0.831, 0.831]	-0.00	.999
		Incorrect	-0.150	0.506	[-1.141, 0.841]	-0.30	.767
		Incorrect–correct	-0.149	0.351	[-0.837, 0.538]	-0.43	.670
Childhood neglect	Male	Correct	-1.838	0.556	[-2.927, -0.749]	-3.31	< .001
		Incorrect	-0.910	0.642	[-2.167, 0.348]	-1.42	.156
		Incorrect–correct	0.929	0.428	[0.090, 1.767]	2.17	.030
	Female	Correct	0.034	0.483	[-0.913, 0.980]	0.07	.944
		Incorrect	0.353	0.569	[-0.762, 1.468]	0.62	.535
		Incorrect–correct	0.319	0.390	[-0.445, 1.083]	0.82	.413

To test whether these sex differences were due to a variance in exposure, we conducted some percentile comparisons. For CTQ neglect, the same raw scores corresponded to highly similar percentiles across genders: at *z* = −1, both males and females were at the floor of the distribution (0%); at *z* = 0, males were at the 62.4th percentile and females at the 64.5th percentile; and at *z* = + 1, males were at the 87.9th percentile and females at the 85.1st percentile. For the CTQ abuse composite, the same raw scores corresponded to slightly higher percentiles in men: at *z* = −1, both genders were again at the floor (0%); at *z* = 0, males were at the 72.5th percentile and females at the 63.4th percentile; and at *z* = + 1, males were at the 92.4th percentile and females at the 84.2nd percentile. To address this, we conducted additional analyses using within-gender standardized CTQ scores, ensuring that comparisons reflect equivalent relative standing within each gender. Critically, the key interaction between trial type, CTQ abuse, and gender remained significant (*p* = .022) in this model. This indicates that the observed gender differences in the ERN x CTQ abuse relationship are robust and not attributable to differences in the distribution of abuse across genders.

In summary, higher CTQ abuse was associated with an enhanced (more negative) ERN primarily in males, whereas ERN amplitude in females showed relatively little variation across levels of CTQ abuse. Higher CTQ neglect was associated with a reduced (less negative) ERN in males, with comparatively smaller changes observed in females. Correct-trial amplitude became less positive (i.e., more negative) as CTQ neglect increased in males, whereas it showed little change in females.

#### 3.3.2 Theta

##### 3.3.2.1 Primary model: Main effects of childhood adversity

We fit a linear mixed-effects model to examine the extent to which CTQ abuse, CTQ neglect, and sex were associated with frontal midline theta power (4–8 Hz), quantified within the fixed 40 ms window centred on the group-level theta peak identified between 250 and 550 ms post-response. Theta power was log-transformed prior to analysis to account for non-normality (Osborne, 2002). We included CTQ abuse (z-scored) and CTQ neglect (z-scored) simultaneously as fixed effects, together with sex, allowing the unique association of each adversity dimension with theta power to be estimated while accounting for their shared variance. Random intercepts were included for laboratory site and participant.

Higher levels of CTQ neglect were associated with lower theta power (see Fig. 6), whereas CTQ abuse was not significantly associated with theta power. Females also exhibited higher theta power than males. Full model results are presented in Table 4.

**Fig. 6.**
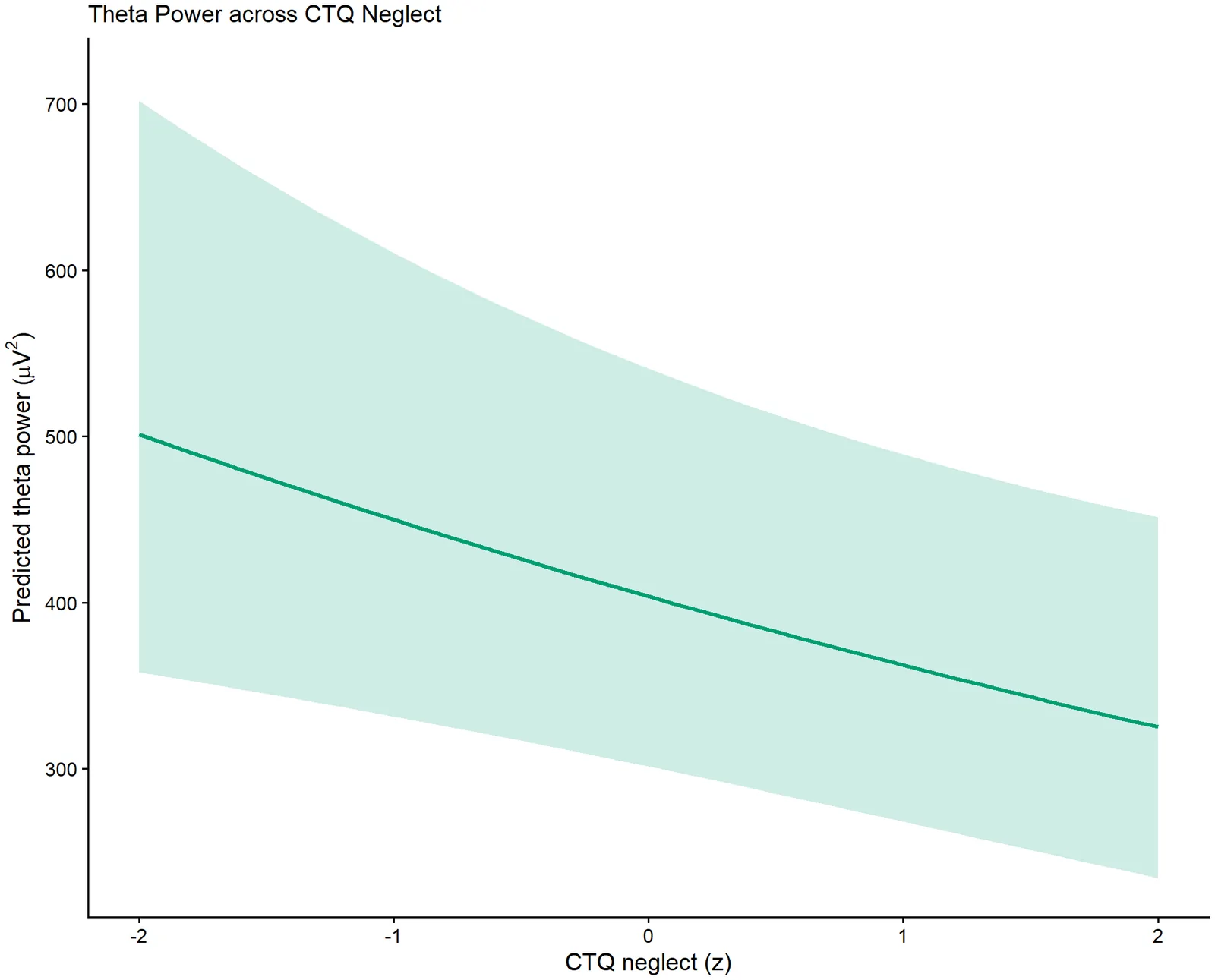
The association between theta power and neglect.

**Table 4.** Linear mixed-effects model results for frontal midline theta.

Model	Predictor	B	SE	95% CI	df	*t*	*p*
Primary model	Intercept	6.001	0.149	[5.672, 6.329]	10.9	40.26	< .001
	Childhood abuse	0.046	0.041	[-0.033, 0.126]	772.7	1.14	.254
	Childhood neglect	-0.108	0.040	[-0.187, -0.029]	772.7	-2.69	.007
	Sex: Female vs. male	0.176	0.062	[0.053, 0.298]	772.4	2.82	.005
Secondary moderation model	Intercept	6.017	0.149	[5.688, 6.345]	11.1	40.28	< .001
	Childhood abuse	0.146	0.081	[-0.012, 0.304]	772.6	1.81	.071
	Childhood neglect	-0.142	0.062	[-0.265, -0.020]	772.9	-2.29	.022
	Sex: Female vs. male	0.165	0.063	[0.042, 0.288]	772.3	2.63	.009
	Childhood abuse × sex	-0.130	0.094	[-0.315, 0.054]	772.7	-1.39	.165
	Childhood neglect × sex	0.044	0.082	[-0.118, 0.206]	772.9	0.54	.592

##### 3.3.2.2 Secondary (exploratory) model: Sex as a moderator

To examine whether the associations between childhood adversity and theta power differed by sex, we fitted a second linear mixed-effects model that extended the primary model by including interactions between sex and CTQ abuse and between sex and CTQ neglect, while retaining the same random-effects structure.

Adding these interaction terms did not improve model fit relative to the primary model. Consistent with the primary model, higher levels of CTQ neglect remained associated with lower theta power, whereas CTQ abuse was not significantly associated with theta power. Females also continued to exhibit higher theta power than males. Neither the CTQ abuse × sex interaction nor the CTQ neglect × sex interaction was significant, indicating that the associations between childhood adversity and theta power did not differ significantly between males and females. Full model results are presented in Table 4.

These effects remained consistent when controlling for anxiety and depression (see Supplementary Table S4). Overall, these findings indicate that CTQ neglect is consistently associated with reduced frontal midline theta power, whereas CTQ abuse shows no reliable main effect. There is limited evidence for a sex-dependent association of childhood abuse, although this effect did not reach statistical significance and did not improve overall model fit. Across models, females exhibited modestly higher theta power than males (see Fig. 6).

#### 3.3.3 Controlling for task performance

To examine whether the EEG findings could be explained by individual differences in task performance, the primary ERN and theta models were repeated with overall error rate included as a covariate. For the ERN, overall error rate was significantly associated with amplitude on correct trials (*b* = -0.63 μV, *SE* = 0.28, *p* = .023). This effect was qualified by a significant interaction between trial type and error rate (*b* = 1.06 μV, *SE* = 0.21, *p *< .001), indicating that overall error rate was associated with the magnitude of the error–correct difference. Importantly, controlling for error rate did not materially alter the adversity findings. The trial type × abuse × gender interaction remained significant (*b* = -1.65 μV, *SE* = 0.64, *p* = .010, 95% CI [-2.91, -0.39]) and comparable with the original model (*b* = -1.72 μV, *SE* = 0.64, *p* = .007, 95% CI [-2.98, -0.46]). The trial type × neglect × gender interaction remained nonsignificant (*b* = 0.60 μV, *SE* = 0.57, *p *= .291, 95% CI [-0.52, 1.73]). For theta power, overall error rate was not a significant predictor (*b* = 0.013, *SE* = 0.031, *p *= .674, 95% CI [-0.047, 0.073]). Its inclusion produced negligible changes in the abuse, neglect, sex, and adversity × sex coefficients. These findings indicate that the observed adversity-related EEG effects were not explained by individual differences in overall task performance.

## 4 Discussion

### 4.1 Summary of findings

Consistent with our hypotheses and the dimensional model of adversity (McLaughlin et al., 2014, 2019), abuse and neglect showed opposing patterns of association with neural measures of performance monitoring. Specifically, higher abuse was associated with enhanced ERN and increased theta power, whereas higher neglect was associated with attenuated ERN and reduced theta. These effects were moderated by sex, such that associations were observed in males but not in females.

### 4.2 ERN and childhood adversity

Prior studies have reported both heightened and blunted ERN responses following adversity. For example, interpersonal stressors such as relational victimization have been associated with increased ERN (Cole et al., 2023), and similar patterns have been observed in smaller samples using cognitive control tasks (Wu et al., 2021). However, other work has found that the relationship between cumulative adversity and a reduced ERN is driven by neglect (Letkiewicz et al., 2023). Such findings are consistent with blunted error-related brain activity reported in those with a history of severe neglect (i.e., institutionalization; Troller-Renfree et al., 2017). Additional studies have reported blunted ERN in the context of depressive symptoms or broader adverse experiences without distinguishing between forms of adversity (Tabachnick et al., 2018; Xue et al., 2017), while some findings remain weak or context dependent in modestly sized samples (Compton et al., 2024). By distinguishing between abuse and neglect in line with the dimensional model of adversity (McLaughlin et al., 2014, 2019) in a large sample, the present findings help reconcile this mixed literature. Specifically, threat-related experiences appear linked to heightened performance monitoring, whereas deprivation-related experiences are associated with attenuated performance monitoring.

Abuse and neglect commonly co-occur in real-world samples (Herrenkohl & Herrenkohl, 2009; Matsumoto et al., 2023), and they were strongly correlated (*r* = .65) in the present sample. This substantial overlap highlights an important challenge for dimensional models of adversity. Separating abuse and neglect can reveal opposing associations that may be obscured when adversity is treated as a unitary construct, but relatively large samples are needed to distinguish their unique effects when the dimensions themselves are strongly related. Their frequent co-occurrence also means that these dimensions may rarely operate in isolation in real-world settings. The present findings, therefore, underscore the importance of examining both their shared and distinct associations with neurocognitive functioning.

### 4.3 Theta and childhood adversity

Our findings also help clarify the literature on theta and childhood adversity, which has rarely distinguished between forms of adversity. At rest, higher early-life home adversity has been linked to greater theta power in childhood, interpreted as reflecting delayed cortical development (Bick et al., 2019). In contrast, task-based studies more often report reduced midline theta following adversity. For example, Kavanaugh et al. (2024) found that recent stressful events were associated with attenuated theta during inhibitory control in children, and institutionalized samples show reduced mediofrontal theta following early neglect, with more normative development following improved caregiving (Buzzell et al., 2020). Consistent with these task-based findings, neglect was associated with attenuated theta during performance monitoring, whereas abuse showed little to no relationship. This dissociation helps reconcile inconsistencies in the literature and supports dimensional models of adversity, which posit distinct neurocognitive adaptations to deprivation and threat.

Frontal midline theta did not significantly differ between correct and incorrect responses or between congruent and incongruent trials. However, frontal midline theta is not specific to errors or conflict. Rather, theta has been proposed to reflect a broader medial frontal process involved in monitoring the need for cognitive control across a range of events, including conflict, novelty, punishment, and error (Cavanagh & Frank, 2014; Cavanagh et al., 2012). Indeed, we observed a significant, albeit small-to-moderate association between ERN amplitudes and error-related theta. Consistent with a broader midfrontal control mechanism, even correct and congruent trials in the flanker task require participants to monitor the stimulus environment, select the appropriate response, and adapt behaviour across trials.

Importantly, the present flanker task used emotionally neutral stimuli, as did the Go/No-Go tasks used by Kavanaugh et al. (2024) and Buzzell et al. (2020). Kavanaugh et al. (2024) used affectively neutral animal images, whereas Buzzell et al. (2020) used letters as Go and No-Go stimuli. It, therefore, remains unclear whether associations between childhood adversity and midfrontal theta differ when cognitive control is required in emotionally salient contexts. This may be particularly relevant following adversity, where associations with cognitive control may differ in the presence of emotionally salient stimuli (e.g., happy vs angry faces). Future research should, therefore, examine how abuse and neglect influence midfrontal theta across both different cognitive control demands and emotionally neutral versus salient contexts. Such an approach would help clarify both the unity and diversity of how adverse childhood experiences influence cognitive control.

### 4.4 Sex differences in associations between adversity and performance monitoring

In exploratory models, we observed consistent moderation by sex across both neural indices, with associations between childhood adversity and performance monitoring emerging in males but not in females. The Adaptive Calibration Model of stress responsivity (Del Giudice et al., 2011) may help explain these findings. According to the Adaptive Calibration Model, stress responsivity represents a form of conditional adaptation in which early environments calibrate neurobiological systems towards context-appropriate behaviour, including heightened vigilance and sensitivity to feedback under threat. From this perspective, males may be more likely to calibrate towards enhanced performance monitoring in high-threat environments (i.e., abuse), reflecting increased sensitivity to mistakes and supporting competitive vigilance. In contrast, females may calibrate towards heightened sensitivity to interpersonal and affective cues, with adaptation expressed in systems supporting social evaluation and emotion regulation rather than performance monitoring. Consistent with this account, early adversity is more often associated with externalizing vigilance in males and internalizing vigilance in females (Jung et al., 2017). Our findings are also consistent with theories of gender socialization in which boys are socialized to value independence, achievement, and competitiveness, whereas girls are socialized to value interpersonal relationships, care, and social connectedness (Ruble et al., 2006). These socialization practices can have downstream consequences for biological systems supporting stress reactivity (Dedovic et al., 2009), in line with the Adaptive Calibration Model. Future research should examine the extent to which adaptations to early adversity in females are preferentially expressed in neural systems supporting socio-affective processes and driven by gender role socialization.

### 4.5 Limitations and future directions

Despite our large sample and dimensional approach to adversity, there are two key limitations. First, overall levels of childhood adversity were relatively low, which may limit generalizability to individuals exposed to more severe or chronic adversity. This limitation is tempered as our findings are consistent with evidence from high-risk samples, where early neglect has been associated with reduced mediofrontal theta and more normative development following improved caregiving environments (Buzzell et al., 2020). Although the physical neglect subscale demonstrated relatively low internal consistency in the present sample, the composite CTQ neglect score used in the primary analyses showed good reliability (α = .83). Nevertheless, future studies should examine emotional and physical neglect separately to determine whether specific dimensions of neglect differentially relate to neural indices of performance monitoring.

Second, the sample consisted exclusively of university students and excluded individuals with physical or psychological disorders. These characteristics may limit generalizability to the broader population, particularly because childhood adversity is associated with socioeconomic disadvantage (O’Connor et al., 2020) and increased risk for physical and mental health problems (Nelson et al., 2020; Nusslock & Miller, 2016). The sample may, therefore, underrepresent individuals with more severe adversity histories and their aftereffects. Our findings suggest that one source of inconsistency in prior research may be the tendency to combine abuse and neglect despite their opposing associations with performance monitoring. However, adversity severity and associated psychopathology may also shape these associations. Future research should, therefore, examine adversity type, severity, and psychopathology in more socioeconomically and educationally diverse samples spanning a broader range of adversity exposure.

Third, this study relied on retrospective reports of adversity which are at best modestly correlated with prospective assessments of adversity (Francis et al., 2023). Future research should examine the extent to which our ERN findings are driven by subjective rather than objective experiences of childhood adversity. Fourth, the cross-sectional design of this study precludes conclusions about developmental trajectories. Sensitive period models suggest that adversity in childhood (<10 years) results in larger frontolimbic cortical volumes and attentional biases towards threat that reverse in adolescence and early adulthood (Weissman et al., 2019; Vannucci et al., 2023). Future longitudinal research should examine the extent to which our performance-monitoring findings reflect the differences between earlier and later development proposed by sensitive period models.

A final limitation is our focus on early performance-monitoring processes, as indexed by the ERN and error-related theta, and did not examine the error positivity (Pe). The Pe is thought to reflect a later stage of error processing associated with conscious error evaluation and can dissociate from the ERN, suggesting that the two components reflect partially independent performance-monitoring processes (Di Gregorio et al., 2018). Notably, the present waveforms suggest that adversity-related differences may extend beyond the ERN time window, although these waveforms were quantified at FCz rather than the more posterior sites at which the Pe is typically maximal. If adversity-related effects also emerge in the Pe, this would suggest that childhood adversity is associated not only with rapid error detection but also with later conscious evaluation of errors. Future research should directly test whether these adversity-related effects extend to the Pe.

### 4.6 Conclusion

Treating childhood adversity as a unitary construct obscures meaningful neurocognitive differences. By separating abuse and neglect, this study demonstrates that these experiences shape distinct patterns of neural activity during performance monitoring and underscores the value of a dimensional approach to childhood adversity.

## Supplementary Material

Supplementary Material

## Data Availability

The study was preregistered prior to data access (https://osf.io/c7pa4/overview?view_only=4d5b4caa319446848f6b1f5e95b7257d) including the study hypotheses and analysis plan. Raw data are not publicly available; however, researchers can apply for access via the CoScience data access process (Paul et al., 2022). All derived data and analysis scripts necessary to reproduce the reported results are publicly available on the Open Science Framework (https://osf.io/c7pa4/overview?view_only=4d5b4caa319446848f6b1f5e95b7257d).

## Footnotes

1 This eligibility criterion was part of the original study design and dataset reported by Paul et al. (2022).

2 The experiment included a rest condition (eyes open/closed, repeated three times), a Go/No-Go task (Go vs. No-Go stimuli), a gambling task (reward/loss and magnitude), an emotional Stroop task (erotic, positive, neutral images), a flanker task (congruence, social evaluation, workload), and an Ultimatum Game (offer fairness).

3 Although the CoScience protocol also included a Go/No-Go task, we selected the Flanker task a priori because prior work suggests that it provides a particularly sensitive measure of error-related brain function, with ERN-related behavioural associations emerging more consistently in the Flanker than the Go/No-Go task (Park et al., 2024; Robins et al., 2026). The present study was preregistered accordingly and Go/No-Go data were not requested or accessed for this secondary analysis.

4 In our CoScience data request, we specified using whichever window (0–50 ms or 0–100 ms) was maximal for ERN. We erroneously specified on the preregistration a 0–50 ms window for ERN quantification. Because the grand-average ERN peaked closer to ~80–100 ms, analyses used the 0–100 ms window.

5 Although preregistration specified residual ERN scores, final analyses modelled correct and incorrect trials within a single mixed-effects model including trial type, retaining trial-level information and accounting for the nested data structure.

## References

[IMAG.a.1439-b1] Bates, D., Mächler, M., Bolker, B., & Walker, S. (2015). Fitting linear mixed-effects models using lme4. Journal of Statistical Software, 67(1), 1–48. 10.18637/jss.v067.i01

[IMAG.a.1439-b2] Becker, M. P. I., Nitsch, A. M., Miltner, W. H. R., & Straube, T. (2014). A single-trial estimation of the feedback-related negativity and its relation to BOLD responses in a time-estimation task. The Journal of Neuroscience, 34(8), 3005–3012. 10.1523/JNEUROSCI.3684-13.2014 PMC6608516 24553940

[IMAG.a.1439-b3] Bernstein, D. P., Stein, J. A., Newcomb, M. D., Walker, E., Pogge, D., Ahluvalia, T., Stokes, J., Handelsman, L., Medrano, M., Desmond, D., & Zule, W. (2003). Development and validation of a brief screening version of the Childhood Trauma Questionnaire. Child Abuse & Neglect, 27(2), 169–190. 10.1016/s0145-2134(02)00541-0 12615092

[IMAG.a.1439-b4] Bick, J., Palmwood, E. N., Zajac, L., Simons, R., & Dozier, M. (2019). Early parenting intervention and adverse family environments affect neural function in middle childhood. Biological Psychiatry, 85(4), 326–335. 10.1016/j.biopsych.2018.09.020 PMC6373871 30447912

[IMAG.a.1439-b5] Botvinick, M. M., Braver, T. S., Barch, D. M., Carter, C. S., & Cohen, J. D. (2001). Conflict monitoring and cognitive control. Psychological Review, 108(3), 624–652. 10.1037/0033-295x.108.3.624 11488380

[IMAG.a.1439-b6] Box, G. E. P., & Cox, D. R. (1964). An analysis of transformations. Journal of the Royal Statistical Society: Series B (Methodological), 26(2), 211–243. 10.1111/j.2517-6161.1964.tb00553.x

[IMAG.a.1439-b7] Buzzell, G. A., Troller-Renfree, S. V., Wade, M., Debnath, R., Morales, S., Bowers, M. E., Zeanah, C. H., Nelson, C. A., & Fox, N. A. (2020). Adolescent cognitive control and mediofrontal theta oscillations are disrupted by neglect: Associations with transdiagnostic risk for psychopathology in a randomized controlled trial. Developmental Cognitive Neuroscience, 43, Article 100777. 10.1016/j.dcn.2020.100777 PMC7150525 32280035

[IMAG.a.1439-b8] Cao, Y., Lizano, P., Li, M., Colic, L., Chand, T., Javaheripour, N., Sun, H., Deng, G., Zhou, X., Long, X., Gong, Q., Qiu, C., Walter, M., & Jia, Z. (2025). Associations among bipolar II depression white matter subgroups, inflammation, symptoms and childhood maltreatment. Nature Mental Health, 3(6), 724–734. 10.1038/s44220-025-00432-4

[IMAG.a.1439-b9] Carter, C. S., Braver, T. S., Barch, D. M., Botvinick, M. M., Noll, D., & Cohen, J. D. (1998). Anterior cingulate cortex, error detection, and the online monitoring of performance. Science, 280(5364), 747–749. 10.1126/science.280.5364.747 9563953

[IMAG.a.1439-b11] Cavanagh, J. F., & Frank, M. J. (2014). Frontal theta as a mechanism for cognitive control. Trends in Cognitive Sciences, 18(8), 414–421. 10.1016/j.tics.2014.04.012 PMC4112145 24835663

[IMAG.a.1439-b12] Cavanagh, J. F., Zambrano-Vazquez, L., & Allen, J. J. B. (2012). Theta lingua franca: A common mid-frontal substrate for action monitoring processes. Psychophysiology, 49(2), 220–238. 10.1111/j.1469-8986.2011.01293.x PMC3262926 22091878

[IMAG.a.1439-b13] Chevalier, N., Hadley, L. V., & Balthrop, K. (2021). Midfrontal theta oscillations and conflict monitoring in children and adults. Developmental Psychobiology, 63(8), e22216. 10.1002/dev.22216 34813101

[IMAG.a.1439-b14] Cohen, M. X. (2014a). Analyzing neural time series data: Theory and practice. MIT Press. 10.7551/mitpress/9609.001.0001

[IMAG.a.1439-b15] Cohen, M. X. (2014b). A neural microcircuit for cognitive conflict detection and signaling. Trends in Neurosciences, 37(9), 480–490. 10.1016/j.tins.2014.06.004 25034536

[IMAG.a.1439-b17] Cole, N., Harvey, M., Myers-Joseph, D., Gilra, A., & Khan, A. G. (2024). Prediction-error signals in anterior cingulate cortex drive task-switching. Nature Communications, 15(1), 7088. 10.1038/s41467-024-51368-9 PMC11330528 39154045

[IMAG.a.1439-b18] Cole, S. L., Mehra, L. M., Cibrian, E., Cummings, E. M., Nelson, B. D., Hajcak, G., & Meyer, A. (2023). Relational victimization prospectively predicts increases in error-related brain activity and social anxiety in children and adolescents across two years. Developmental Cognitive Neuroscience, 61, 101252. 10.1016/j.dcn.2023.101252 PMC10199180 37182336

[IMAG.a.1439-b19] Compton, R. J., Shudrenko, D., Ng, E., Mann, K., & Turdukulov, E. (2024). Adversity and error‐monitoring: Effects of emotional context. Psychophysiology, 61(11), Article e14644. 10.1111/psyp.14644 38963045

[IMAG.a.1439-b20] DeCross, S. N., Sambrook, K. A., Sheridan, M. A., Tottenham, N., & McLaughlin, K. A. (2022). Dynamic alterations in neural networks supporting aversive learning in children exposed to trauma: Neural mechanisms underlying psychopathology. Biological Psychiatry, 91(7), 667–675. 10.1016/j.biopsych.2021.09.013 PMC8917987 34916067

[IMAG.a.1439-b21] Dedovic, K., Wadiwalla, M., Engert, V., & Pruessner, J. C. (2009). The role of sex and sex socialization in stress reactivity. Developmental Psychology, 45(1), 45–55. 10.1037/a0014433 19209989

[IMAG.a.1439-b23] Del Giudice, M., Ellis, B. J., & Shirtcliff, E. A. (2011). The Adaptive Calibration Model of stress responsivity. Neuroscience & Biobehavioral Reviews, 35(7), 1562–1592. 10.1016/j.neubiorev.2010.11.007 PMC3068241 21145350

[IMAG.a.1439-b24] Di Gregorio, F., Maier, M. E., & Steinhauser, M. (2018). Errors can elicit an error positivity in the absence of an error negativity: Evidence for independent systems of human error monitoring. NeuroImage, 172, 427–436. 10.1016/j.neuroimage.2018.01.081 29409999

[IMAG.a.1439-b25] Eriksen, B. A., & Eriksen, C. W. (1974). Effects of noise letters upon the identification of a target letter in a nonsearch task. Perception & Psychophysics, 16(1), 143–149. 10.3758/bf03203267

[IMAG.a.1439-b26] Faul, F., Erdfelder, E., Lang, A.-G., & Buchner, A. (2007). G*Power 3: A flexible statistical power analysis program for the social, behavioral, and biomedical sciences. Behavior Research Methods, 39(2), 175–191. 10.3758/BF03193146 17695343

[IMAG.a.1439-b27] Felitti, V. J., Anda, R. F., Nordenberg, D., Williamson, D. F., Spitz, A. M., Edwards, V., Koss, M. P., & Marks, J. S. (2019). Relationship of childhood abuse and household dysfunction to many of the leading causes of death in adults: The Adverse Childhood Experiences (ACE) study. American Journal of Preventive Medicine, 56(6), 774–786. 10.1016/j.amepre.2019.04.001 31104722

[IMAG.a.1439-b28] Francis, E. R., Tsaligopoulou, A., Stock, S. E., Pingault, J.‐B., & Baldwin, J. R. (2023). Subjective and objective experiences of childhood adversity: A meta‐analysis of their agreement and relationships with psychopathology. Journal of Child Psychology and Psychiatry, 64(8), 1185–1199. 10.1111/jcpp.13803 PMC10617978 37186463

[IMAG.a.1439-b30] Gehring, W. J., Goss, B., Coles, M. G., Meyer, D. E., & Donchin, E. (1993). A neural system for error detection and compensation. Psychological Science, 4(6), 385–390. 10.1111/j.1467-9280.1993.tb00586.x

[IMAG.a.1439-b31] Goetschius, L. G., Hein, T. C., McLanahan, S. S., Brooks-Gunn, J., McLoyd, V. C., Dotterer, H. L., Lopez-Duran, N., Mitchell, C., Hyde, L. W., Monk, C. S., & Beltz, A. M. (2020). Association of childhood violence exposure with adolescent neural network density. JAMA Network Open, 3(9), e2017850. 10.1001/jamanetworkopen.2020.17850 PMC7512058 32965498

[IMAG.a.1439-b32] Gruhn, M., Miller, A. B., Eisenlohr-Moul, T. A., Martin, S., Clayton, M. G., Giletta, M., Hastings, P. D., Nock, M. K., Rudolph, K. D., Slavich, G. M., Prinstein, M. J., & Sheridan, M. A. (2024). Threat exposure moderates associations between neural and physiological indices of emotion reactivity in adolescent females. Psychoneuroendocrinology, 159, 106405. 10.1016/j.psyneuen.2023.106405 PMC11034839 37812939

[IMAG.a.1439-b34] Herrenkohl, R. C., & Herrenkohl, T. I. (2009). Assessing a child’s experience of multiple maltreatment types: Some unfinished business. Journal of Family Violence, 24(7), 485–496. 10.1007/s10896-009-9247-2 PMC2818150 20161642

[IMAG.a.1439-b35] Holroyd, C. B., & Coles, M. G. H. (2002). The neural basis of human error processing: Reinforcement learning, dopamine, and the error-related negativity. Psychological Review, 109(4), 679–709. 10.1037/0033-295x.109.4.679 12374324

[IMAG.a.1439-b36] Holroyd, C. B., & Verguts, T. (2021). The best laid plans: Computational principles of anterior cingulate cortex. Trends in Cognitive Sciences, 25(4), 316–329. 10.1016/j.tics.2021.01.008 33593641

[IMAG.a.1439-b37] Hyvärinen, A., & Oja, E. (2000). Independent component analysis: Algorithms and applications. Neural Networks, 13(4–5), 411–430. 10.1016/s0893-6080(00)00026-5 10946390

[IMAG.a.1439-b38] Imburgio, M. J., Banica, I., Hill, K. E., Weinberg, A., Foti, D., & MacNamara, A. (2020). Establishing norms for error-related brain activity during the arrow Flanker task among young adults. NeuroImage, 213, 116694. 10.1016/j.neuroimage.2020.116694 PMC7197955 32142881

[IMAG.a.1439-b40] Jas, M., Engemann, D. A., Bekhti, Y., Raimondo, F., & Gramfort, A. (2017). Autoreject: Automated artifact rejection for MEG and EEG data. NeuroImage, 159, 417–429. 10.1016/j.neuroimage.2017.06.030 PMC7243972 28645840

[IMAG.a.1439-b41] Jenness, J. L., Peverill, M., Miller, A. B., Heleniak, C., Robertson, M. M., Sambrook, K. A., Sheridan, M. A., & McLaughlin, K. A. (2021). Alterations in neural circuits underlying emotion regulation following child maltreatment: A mechanism underlying trauma-related psychopathology. Psychological Medicine, 51(11), 1880–1889. 10.1017/S0033291720000641 PMC7541399 32252835

[IMAG.a.1439-b44] Jung, H., Herrenkohl, T. I., Lee, J. O., Hemphill, S. A., Heerde, J. A., & Skinner, M. L. (2017). Gendered pathways from child abuse to adult crime through internalizing and externalizing behaviors in childhood and adolescence. Journal of Interpersonal Violence, 32(18), 2724–2750. 10.1177/0886260515596146 PMC4991959 26264725

[IMAG.a.1439-b45] Kałamała, P., Szewczyk, J., Senderecka, M., & Wodniecka, Z. (2017). Flanker task with equiprobable congruent and incongruent conditions does not elicit the conflict N2. Psychophysiology, 55(2), e12980. 10.1111/psyp.12980 28845513

[IMAG.a.1439-b46] Kavanaugh, B. C., Parade, S., Seifer, R., McLaughlin, N. C. R., Tirrell, E., Festa, E. K., Oberman, L. M., Novick, A. M., Carpenter, L. L., & Tyrka, A. R. (2024). Childhood stress, sex, and cognitive control: Midline theta power. Journal of Psychiatric Research, 169, 298–306. 10.1016/j.jpsychires.2023.11.046 PMC10997405 38070470

[IMAG.a.1439-b48] Klinitzke, G., Romppel, M., Häuser, W., Brähler, E., & Glaesmer, H. (2012). Die Deutsche version des Childhood Trauma Questionnaire (CTQ)—Psychometrische eigenschaften in einer bevölkerungsrepräsentativen stichprobe [The German version of the Childhood Trauma Questionnaire (CTQ)—Psychometric characteristics in a representative sample of the general population]. PPmP: Psychotherapie Psychosomatik Medizinische Psychologie, 62(2), 47–51. 10.1055/s-0031-1295495 22203470

[IMAG.a.1439-b49] Kuznetsova, A., Brockhoff, P. B., & Christensen, R. H. B. (2017). lmerTest package: Tests in linear mixed effects models. Journal of Statistical Software, 82(13), 1–26. 10.18637/jss.v082.i13

[IMAG.a.1439-b50] Lackner, C. L., Santesso, D. L., Dywan, J., O’Leary, D. D., Wade, T. J., & Segalowitz, S. J. (2018). Adverse childhood experiences are associated with self-regulation and the magnitude of the error-related negativity difference. Biological Psychology, 132, 244–251. 10.1016/j.biopsycho.2018.01.006 29309827

[IMAG.a.1439-b51] Lenth, R. V. (2024). emmeans: Estimated marginal means, aka least-squares means [R package]. https://CRAN.R-project.org/package=emmeans

[IMAG.a.1439-b52] Letkiewicz, A. M., Spring, J. D., Li, L. Y., Weinberg, A., & Shankman, S. A. (2023). Childhood trauma predicts blunted error monitoring in adulthood: An event-related potential study. Cognitive, Affective & Behavioral Neuroscience, 23(2), 427–439. 10.3758/s13415-023-01061-z PMC10065923 36653556

[IMAG.a.1439-b54] Lynch, K. S., & Widom, C. S. (2022). Childhood maltreatment and cognitive functioning in middle adulthood. Child Abuse & Neglect, 132, 105791. 10.1016/j.chiabu.2022.105791 35907334

[IMAG.a.1439-b55] Machlin, L., Sheridan, M. A., Lurie, L. A., Kasparek, S. W., Kim, S. G., Peverill, M., France, J. M., Robertson, M. M., Jovanovic, T., Lengua, L. J., & McLaughlin, K. A. (2024). Alterations in fear learning as a mechanism linking childhood exposure to violence with PTSD symptoms: A longitudinal study. Psychological Medicine, 54(12), 3389–3397. 10.1017/s0033291724001569 PMC11983661 39245946

[IMAG.a.1439-b56] Matsumoto, M., Piersiak, H. A., Letterie, M. C., & Humphreys, K. L. (2023). Population-based estimates of associations between child maltreatment types: A meta-analysis. Trauma, Violence, & Abuse, 24(2), 487–496. 10.1177/15248380211030502 34275382

[IMAG.a.1439-b58] McLaughlin, K. A. (2016). Future directions in childhood adversity and youth psychopathology. Journal of Clinical Child & Adolescent Psychology, 45(3), 361–382. 10.1080/15374416.2015.1110823 PMC4837019 26849071

[IMAG.a.1439-b59] McLaughlin, K. A., Sheridan, M. A., & Lambert, H. K. (2014). Childhood adversity and neural development: Deprivation and threat as distinct dimensions of early experience. Neuroscience & Biobehavioral Reviews, 47, 578–591. 10.1016/j.neubiorev.2014.10.012 PMC4308474 25454359

[IMAG.a.1439-b60] McLaughlin, K. A., Weissman, D., & Bitrán, D. (2019). Childhood adversity and neural development: A systematic review. Annual Review of Developmental Psychology, 1(1), 277–312. 10.1146/annurev-devpsych-121318-084950 PMC7243625 32455344

[IMAG.a.1439-b61] National Institute for Health and Care Excellence. (2017). Child abuse and neglect (NICE Guideline No. 76). https://www.nice.org.uk/guidance/ng76

[IMAG.a.1439-b62] Nelson, C. A., Bhutta, Z. A., Harris, N. B., Danese, A., & Samara, M. (2020). Adversity in childhood is linked to mental and physical health throughout life. BMJ, 371, m3048. 10.1136/bmj.m3048 PMC7592151 33115717

[IMAG.a.1439-b63] Nusslock, R., & Miller, G. E. (2016). Early-life adversity and physical and emotional health across the lifespan: A neuroimmune network hypothesis. Biological Psychiatry, 80(1), 23–32. 10.1016/j.biopsych.2015.05.017 PMC4670279 26166230

[IMAG.a.1439-b64] O’Connor, M., Slopen, N., Becares, L., Burgner, D., Williams, D. R., & Priest, N. (2020). Inequalities in the distribution of childhood adversity from birth to 11 years. Academic Pediatrics, 20(5), 609–618. 10.1016/j.acap.2019.12.004 31841661

[IMAG.a.1439-b65] Olvet, D. M., & Hajcak, G. (2009). The stability of error-related brain activity with increasing trials. Psychophysiology, 46(5), 957–961. 10.1111/j.1469-8986.2009.00848.x 19558398

[IMAG.a.1439-b66] Osborne, J. (2002). Notes on the use of data transformations. Practical Assessment, Research, and Evaluation, 8(1), 6. 10.7275/4vng-5608

[IMAG.a.1439-b67] Park, B., Holbrook, A., Lutz, M. C., Baldwin, S. A., Larson, M. J., & Clayson, P. E. (2024). Task-specific relationships between error-related ERPs and behavior: Flanker, Stroop, and Go/Nogo tasks. International Journal of Psychophysiology, 204, 112409. 10.1016/j.ijpsycho.2024.112409 39121995

[IMAG.a.1439-b68] Paul, K., Short, C. A., Beauducel, A., Carsten, H. P., Härpfer, K., Hennig, J., Hewig, J., Hildebrandt, A., Kührt, C., Mueller, E. M., Munk, A., Osinsky, R., Porth, E., Riesel, A., Rodrigues, J., Scheffel, C., Stahl, J., Strobel, A., & Wacker, J. (2022). The methodology and dataset of the Coscience EEG-Personality Project: A large-scale, multi-laboratory project grounded in cooperative forking paths analysis. Personality Science, 3(1), e7177. 10.5964/ps.7177

[IMAG.a.1439-b69] Pechtel, P., & Pizzagalli, D. A. (2011). Effects of early life stress on cognitive and affective function: An integrated review of human literature. Psychopharmacology, 214(1), 55–70. 10.1007/s00213-010-2009-2 PMC3050094 20865251

[IMAG.a.1439-b70] R Core Team. (2025). R: A language and environment for statistical computing (Version 4.5.0) [Computer software]. R Foundation for Statistical Computing. https://www.R-project.org/

[IMAG.a.1439-b72] Rivera, P. M., Fincham, F. D., & Bray, B. C. (2017). Latent classes of maltreatment: A systematic review and critique. Child Maltreatment, 23(1), 3–24. 10.1177/1077559517728125 PMC5614894 28875728

[IMAG.a.1439-b73] Robins, E. M., Zhou, Z., Huang, C., Angus, D. J., Sedikides, C., & Kelley, N. J. (2026). Narcissism is associated with blunted error‐related brain activity. Journal of Personality, 94(4), 741–757. 10.1111/jopy.70036 PMC13359291 41388595

[IMAG.a.1439-b74] Ruble, D. N., Martin, C. L., & Berenbaum, S. A. (2006). Gender development. In N. Eisenberg, W. Damon, & R. M. Lerner (Eds.), Handbook of child psychology: Social, emotional, and personality development (6th ed., pp. 858–932). John Wiley & Sons, Inc. 10.1002/9780470147658.chpsy0314

[IMAG.a.1439-b76] Schäfer, J. L., McLaughlin, K. A., Manfro, G. G., Pan, P., Rohde, L. A., Miguel, E. C., Simioni, A., Hoffmann, M. S., & Salum, G. A. (2023). Threat and deprivation are associated with distinct aspects of cognition, emotional processing, and psychopathology in children and adolescents. Developmental Science, 26(1), 1–12. 10.1111/desc.13267 PMC10028496 35417607

[IMAG.a.1439-b77] Sheridan, M. A., Peverill, M., Finn, A. S., & McLaughlin, K. A. (2017). Dimensions of childhood adversity have distinct associations with neural systems underlying executive functioning. Development and Psychopathology, 29(5), 1777–1794. 10.1017/S0954579417001390 PMC5733141 29162183

[IMAG.a.1439-b80] Tabachnick, A. R., Valadez, E. A., Palmwood, E. N., Zajac, L., Simons, R. F., & Dozier, M. (2018). Depressive symptoms and error‐related brain activity in CPS‐referred children. Psychophysiology, 55(11), 1–13. 10.1111/psyp.13211 PMC6193840 30094846

[IMAG.a.1439-b81] Teicher, M. H., Samson, J. A., Anderson, C. M., & Ohashi, K. (2016). The effects of childhood maltreatment on brain structure, function and connectivity. Nature Reviews Neuroscience, 17(10), 652–666. 10.1038/nrn.2016.111 27640984

[IMAG.a.1439-b82] Troller-Renfree, S., Zeanah, C. H., Nelson, C. A., & Fox, N. A. (2017). Neural and cognitive factors influencing the emergence of psychopathology: Insights from the Bucharest early intervention project. Child Development Perspectives, 12(1), 28–33. 10.1111/cdep.12251 PMC5844478 29531577

[IMAG.a.1439-b83] Umemoto, A., HajiHosseini, A., Yates, M. E., & Holroyd, C. B. (2017). Reward-based contextual learning supported by anterior cingulate cortex. Cognitive, Affective, & Behavioral Neuroscience, 17(3), 642–651. 10.3758/s13415-017-0502-3 28236171

[IMAG.a.1439-b84] Vannucci, A., Fields, A., Hansen, E., Katz, A., Kerwin, J., Tachida, A., Martin, N., & Tottenham, N. (2023). Interpersonal early adversity demonstrates dissimilarity from early socioeconomic disadvantage in the course of human brain development: A meta-analysis. Neuroscience & Biobehavioral Reviews, 150, 105210. 10.1016/j.neubiorev.2023.105210 PMC10247458 37141961

[IMAG.a.1439-b86] Wang, S., Zhou, N., Cao, H., & Lin, X. (2026). Linking deprivation in early childhood with academic performance in middle adolescence through cognitive ability in middle childhood: Nuance by specific cognitive component and heterogeneity by child negative emotionality. Development and Psychopathology, 38(2), 882–898. 10.1017/S0954579425100813 PMC12825954 41131884

[IMAG.a.1439-b87] Weissman, D. G., Bitran, D., Miller, A. B., Schaefer, J. D., Sheridan, M. A., & McLaughlin, K. A. (2019). Difficulties with emotion regulation as a transdiagnostic mechanism linking child maltreatment with the emergence of psychopathology. Development and Psychopathology, 31(3), 899–915. 10.1017/S0954579419000348 PMC6620140 30957738

[IMAG.a.1439-b88] Weissman, D. G., Jenness, J. L., Colich, N. L., Miller, A. B., Sambrook, K. A., Sheridan, M. A., & McLaughlin, K. A. (2020). Altered neural processing of threat-related information in children and adolescents exposed to violence: A transdiagnostic mechanism contributing to the emergence of psychopathology. Journal of the American Academy of Child & Adolescent Psychiatry, 59(11), 1274–1284. 10.1016/j.jaac.2019.08.471 PMC7048648 31473292

[IMAG.a.1439-b89] Wickham, H. (2016). Data analysis. In ggplot2: Elegant graphics for data analysis (pp. 189–201). Springer. 10.1007/978-3-319-24277-4_9

[IMAG.a.1439-b90] Wickham, H., Averick, M., Bryan, J., Chang, W., McGowan, L. D., François, R., Grolemund, G., Hayes, A., Henry, L., Hester, J., Kuhn, M., Pedersen, T. L., Miller, E., Bache, S. M., Müller, K., Ooms, J., Robinson, D., Seidel, D. P., Spinu, V.,… Yutani, H. (2019). Welcome to the tidyverse. Journal of Open Source Software, 4(43), 1686. 10.21105/joss.01686

[IMAG.a.1439-b91] Wickham, H., & Seidel, D. (2022). scales: Scale functions for visualization [R package]. https://CRAN.R-project.org/package=scales

[IMAG.a.1439-b92] Wong, W. M., Zhang, B., Foley, D., & Nikulina, V. (2025). Childhood maltreatment impacts cognitive function in emerging adulthood: A systematic review and meta-analysis. Psychological Reports. 10.1177/00332941251411294 41467328

[IMAG.a.1439-b93] Wu, J., Liu, Y., Fang, H., Qin, S., Kohn, N., & Duan, H. (2021). The relationship between childhood stress and distinct stages of dynamic behavior monitoring in adults: Neural and behavioral correlates. Social Cognitive and Affective Neuroscience, 16(9), 937–949. 10.1093/scan/nsab041 PMC8421694 33830244

[IMAG.a.1439-b94] Xue, J.-M., Lin, P.-Z., Sun, J.-W., & Cao, F.-L. (2017). Disrupted executive function and aggression in individuals with a history of adverse childhood experiences: An event-related potential study. Journal of Nervous and Mental Disease, 205(12), 942–951. 10.1097/NMD.0000000000000743 28976406

