# Supplementary material for: Opposing effects of childhood abuse and neglect on neural performance monitoring
Source: Imaging Neurosci (Camb). 2026 Nov 4;4:IMAG.a.1439. doi: 10.1162/IMAG.a.1439 (PMC13736806; doi:10.1162/IMAG.a.1439)
Supplement: Supplementary file 1 [file IMAG.a.1439_supp.pdf]

Supplementary Materials for:

Opposing Effects of Childhood Abuse and Neglect on Neural Performance Monitoring

Esther M. Robins[1], Zhiwei Zhou[1], Douglas J. Angus[2], Dennis Golm[1], Nicholas J. Kelley[1]

[1]School of Psychology, University of Southampton

[2]School of Psychology, Bond University, Australia

Supplementary Analysis

Table S1

Bayesian Linear Mixed-Effects Model: ERN

	Primary model		***Secondary model***	
**Fixed effect**	***B (SD)***	**95% CrI**	***B (SD)***	**95% CrI**
Intercept	2.64 (0.43)	[1.79, 3.51]	2.99 (0.42)	[2.18, 3.83]
Trial type: Incorrect	-3.65 (0.29)	[-4.23, -3.07]	-3.84 (0.30)	[-4.42, -3.25]
Childhood abuse	0.78 (0.36)	[0.08, 1.47]	2.86 (0.70)	[1.52, 4.25]
Childhood neglect	-0.60 (0.36)	[-1.29, 0.13]	-1.74 (0.55)	[-2.81, -0.66]
Sex: Female	-1.79 (0.56)	[-2.86, -0.67]	-2.04 (0.55)	[-3.13, -0.94]
Trial type: Incorrect × Childhood abuse	-0.61 (0.29)	[-1.18, -0.04]	-1.83 (0.52)	[-2.85, -0.80]
Trial type: Incorrect × Childhood neglect	0.48 (0.29)	[-0.09, 1.08]	0.89 (0.41)	[0.09, 1.73]
Trial type: Incorrect × Sex: Female	0.90 (0.42)	[0.06, 1.71]	1.02 (0.43)	[0.17, 1.84]
Childhood abuse × Sex: Female			-2.80 (0.81)	[-4.43, -1.28]
Trial type: Incorrect × Childhood abuse × Sex: Female			1.67 (0.63)	[0.45, 2.93]
Sex: Female × Childhood neglect			1.75 (0.71)	[0.32, 3.12]
Trial type: Incorrect × Sex: Female × Childhood neglect			-0.58 (0.55)	[-1.66, 0.51]

Note. B values are posterior means, with posterior standard deviations in parentheses, and are reported in μV. CrI = credible interval. Correct trials and males constitute the reference categories. Continuous predictors were standardised. Childhood abuse and neglect were entered simultaneously. Both models included random intercepts for participant and laboratory. The secondary model additionally included adversity × sex and trial type × adversity × sex interaction terms.

Table S2

Bayesian Linear Mixed-Effects Model: Theta

	Primary model		***Secondary model***	
**Fixed effect**	**B (SD)**	**95% CrI**	**B (SD)**	**95% CrI**
Intercept	5.998 (0.184)	[5.644, 6.396]	6.032 (0.180)	[5.671, 6.391]
Childhood abuse	0.050 (0.039)	[-0.026, 0.125]	0.137 (0.082)	[-0.018, 0.301]
Childhood neglect	-0.109 (0.041)	[-0.187, -0.028]	-0.141 (0.060)	[-0.260, -0.024]
Sex: Female	0.165 (0.060)	[0.051, 0.284]	0.173 (0.063)	[0.045, 0.294]
Childhood abuse × Sex: Female			-0.118 (0.095)	[-0.307, 0.060]
Childhood neglect × Sex: Female			0.040 (0.081)	[-0.118, 0.193]

Note. B values are posterior means, with posterior standard deviations in parentheses. Estimates are reported for log-transformed theta power expressed in μV². CrI = credible interval. Males constitute the reference category. Childhood abuse and childhood neglect were standardised and entered simultaneously. Both models included random intercepts for participant and laboratory. The secondary model additionally included the childhood abuse × sex and childhood neglect × sex interaction terms.

Table S3

Frequentist Models Controlling for Anxiety and Depression: ERN

	Primary model				Secondary model			
**Fixed effect**	**B (SE)**	**95% CI**	***t***	***p***	**B (SE)**	**95% CI**	***t***	***p***
Intercept	2.69 (0.40)	[1.90, 3.48]	6.72	< .001	3.04 (0.41)	[2.23, 3.84]	7.43	< .001
Trial type: Incorrect	-3.66 (0.30)	[-4.24, -3.07]	-12.26	< .001	-3.85 (0.31)	[-4.46, -3.25]	-12.57	< .001
Childhood abuse	0.63 (0.37)	[-0.09, 1.36]	1.72	.086	2.82 (0.72)	[1.42, 4.23]	3.95	< .001
Childhood neglect	-0.69 (0.36)	[-1.41, 0.02]	-1.92	.056	-1.97 (0.56)	[-3.06, -0.87]	-3.53	< .001
Sex: Female	-1.92 (0.59)	[-3.07, -0.77]	-3.28	.001	-2.13 (0.58)	[-3.28, -0.98]	-3.65	< .001
Anxiety	-0.05 (0.31)	[-0.67, 0.57]	-0.16	.874	-0.07 (0.31)	[-0.68, 0.54]	-0.22	.823
Depression	0.67 (0.31)	[0.05, 1.28]	2.14	.033	0.68 (0.31)	[0.08, 1.29]	2.21	.028
Trial type × Childhood abuse	-0.61 (0.29)	[-1.18, -0.04]	-2.09	.037	-1.86 (0.54)	[-2.92, -0.81]	-3.47	< .001
Trial type × Childhood neglect	0.47 (0.28)	[-0.09, 1.03]	1.65	.098	0.92 (0.43)	[0.09, 1.76]	2.16	.031
Trial type × Sex	0.91 (0.43)	[0.07, 1.76]	2.12	.034	1.03 (0.43)	[0.19, 1.88]	2.39	.017
Childhood abuse × Sex					-3.00 (0.83)	[-4.62, -1.37]	-3.62	< .001
Childhood neglect × Sex					1.97 (0.74)	[0.53, 3.41]	2.68	.007
Trial type × Childhood abuse × Sex					1.72 (0.64)	[0.46, 2.97]	2.68	.007
Trial type × Childhood neglect × Sex					-0.61 (0.58)	[-1.74, 0.53]	-1.05	.294

Note. B and SE values are reported in μV. Correct trials and males constitute the reference categories. Continuous predictors were standardised. Childhood abuse and neglect were entered simultaneously. Both models controlled for anxiety and depression and included random intercepts for participant and laboratory. The primary model included trial type × adversity and trial type × sex interactions. The secondary model additionally included adversity × sex and trial type × adversity × sex interactions.

Table S4

Frequentist Models Controlling for Anxiety and Depression: Theta

			**Primary model**			***Secondary model***		
**Fixed effect**	**B (SE)**	**B (SE)**	**95% CI**	**95% CI**	***t***	***t***	***p***	***p***
Intercept	6.011 (0.150)	6.026 (0.150)	[5.682, 6.340]	[5.697, 6.355]	40.18	40.18	< .001	< .001
Childhood abuse	0.040 (0.041)	0.137 (0.081)	[-0.042, 0.121]	[-0.022, 0.296]	0.96	1.69	.340	.092
Childhood neglect	-0.113 (0.040)	-0.149 (0.063)	[-0.192, -0.033]	[-0.272, -0.026]	-2.78	-2.38	.005	.018
Sex: Female	0.155 (0.066)	0.146 (0.066)	[0.025, 0.284]	[0.016, 0.275]	2.35	2.20	.019	.028
Anxiety	0.030 (0.036)	0.027 (0.036)	[-0.042, 0.101]	[-0.044, 0.099]	0.81	0.74	.418	.457
Depression	0.015 (0.036)	0.015 (0.036)	[-0.055, 0.085]	[-0.056, 0.085]	0.42	0.41	.672	.681
Childhood abuse × Sex: Female		-0.128 (0.094)		[-0.312, 0.057]		-1.36		.174
Childhood neglect × Sex: Female		0.049 (0.083)		[-0.113, 0.211]		0.59		.554

Note. B values represent regression coefficients for log-transformed theta power expressed in μV^2, with standard errors in parentheses. Males constitute the reference category. Continuous predictors were standardised. Childhood abuse and neglect were entered simultaneously. Both models controlled for anxiety and depression and included random intercepts for participant and laboratory. The secondary model additionally included the childhood abuse × sex and childhood neglect × sex interaction terms.
